# Creep Mechanism and Microstructure Evolution of a Directionally Solidified Ni-Based Superalloy with Different Orientations at 850 °C

**DOI:** 10.3390/ma18071540

**Published:** 2025-03-28

**Authors:** Anping Long, Jiangying Xiong, Bing Wei, Gaoxiang Zhang, Ganjiang Feng, Jianzheng Guo, Rutie Liu

**Affiliations:** 1 State Key Laboratory of Powder Metallurgy, Central South University, Changsha 410083, China; 193302073@csu.edu.cn (A.L.); 203302086@csu.edu.cn (J.X.); 243392006@csu.edu.cn (G.Z.); fenggj@wedge.com.cn (G.F.); 218062@csu.edu.cn (J.G.); 2 Wedge Central South Research Institute Co., Ltd., Shenzhen 518045, China; weibing0522@126.com

**Keywords:** creep mechanisms, microstructure evolution, directionally solidified superalloys, orientations

## Abstract

The creep properties of directionally solidified superalloys are largely influenced by the degradation rate of the γ/γ’ microstructure and the dislocation motion, which exhibit distinct mechanisms under varying temperature and stress conditions. In this study, the creep deformation mechanisms and microstructural evolution of a directionally solidified nickel-based superalloy in the longitudinal (L) and transverse (T) orientations at 850 °C are comprehensively investigated. Creep testing and characterization of the dislocation structure revealed superior creep properties in the L direction compared to the T direction. The creep mechanism in the L direction involves the activation of multiple {111}<110> slip systems, shearing the γ’ precipitates through antiphase boundaries (APBs). Conversely, the creep mechanism in the T direction involves the activation of {111}<112> slip systems, shearing the γ’ precipitates through a superlattice intrinsic stacking fault (SISF) and forming slip bands inclined to the stress axis. Aluminum was identified as the controlling element for the γ’ rafting. The longitudinal specimens exhibited P-type rafting due to the activation of multiple slip systems and sufficient plastic strain flow from the dislocation motion. In contrast, the transverse specimens show little rafting due to limited slip system activation. These findings can serve as a reference for better understanding the anisotropy of directionally solidified superalloys and provide a basis for their broader application.

## 1. Introduction

Directionally solidified nickel-based superalloys, such as CM247LC, GTD111, DZ125, DZ22, and IC10, are widely used in aero-engines and gas turbines due to their excellent high-temperature creep properties and cost-effectiveness [1,2,3,4,5]. Their remarkable high-temperature creep strength is attributed to the strengthening effect of the γ/γ’ microstructure and the grain boundary-free characteristics along the growth direction [6]. The creep properties of directionally solidified superalloys are largely constrained by the degradation rate of the γ/γ’ microstructure and the motion of dislocations within the microstructure [7]. Temperature and applied load significantly influence dislocation motion, and directionally solidified superalloys exhibit different creep mechanisms under low (≈850 °C), medium (≈980 °C), and high (≈1070 °C) temperature conditions [8,9,10].

The creep mechanisms and microstructural evolution of directionally solidified superalloys under different conditions have been extensively studied. For example, Zhang P. et al. [11] found that the creep of directionally solidified M4706 alloy at 900 °C is primarily dominated by dislocation climb and a<001> dislocation shearing within the γ’ precipitates. Guo J. et al. [12] discovered that the creep deformation mechanism of directionally solidified DZ417G alloy is γ’-shearing under low-temperature/high-stress conditions and dislocation climb under high-temperature/low-stress conditions. Tetsu I. et al. [13] investigated the driving force for γ’ rafting in directionally solidified superalloys from the perspective of lattice mismatch and total mechanical energy. Due to the elastic mismatch, the [001] rafting structure exhibits elastic anisotropy, making it more stable under external stress than the [100] or [010] rafting structures. Cao L. et al. [14] revealed significant differences in rafting behavior between uniaxial and multiaxial creep processes, providing new insights into the microstructural evolution of nickel-based superalloys during creep.

Notably, directionally solidified superalloys play a crucial role not only in the manufacture of turbine blades but also in the production of high-temperature guide vanes. But there are still many challenges to be addressed in order to further expand their applications, such as the mechanical limitations brought by anisotropy [15,16,17] and the performance advantages of higher-generation single-crystal alloys [18,19,20]. The influence of different orientations on the creep properties of single-crystal alloys has been extensively studied. For example, Wang G. et al. [21] found that in [111]-oriented IC9 single-crystal alloy, dislocations first slip and multiply in the γ phase to form a dislocation network during creep. Subsequently, some dislocations cut into the γ’ precipitates, forming Kear–Wilsdorf locks. Finally, the dislocation network is disrupted, and dislocations cut into the γ’ precipitates in pairs, forming antiphase boundaries. Sass’s research on CMSX-4 alloy revealed that the creep strength exhibits anisotropy at 850 °C, with the creep strength decreasing in the order of [001], [011], and [111] orientations [22]. However, the deformation mechanisms and microstructural evolution in directions perpendicular to the growth direction have rarely attracted attention.

Although the application of directionally solidified superalloys generally takes advantage of the growth direction, in complex structural components such as guide vanes, it is not possible to ensure that whole parts are oriented in the optimal direction, and the load in some local areas may even be perpendicular to the growth direction. Currently, there are few studies on the creep deformation mechanisms of directionally solidified nickel-based superalloys in directions other than the growth direction. This work comparatively investigates the creep properties, deformation mechanisms, and microstructural evolution of nickel-based superalloys in the solidification direction and perpendicular to the solidification direction, which is of great significance for the design and application of directionally solidified nickel-based superalloy components in the hot sections of aero-engines and gas turbines.

## 2. Materials and Methods

**Figure 1:**
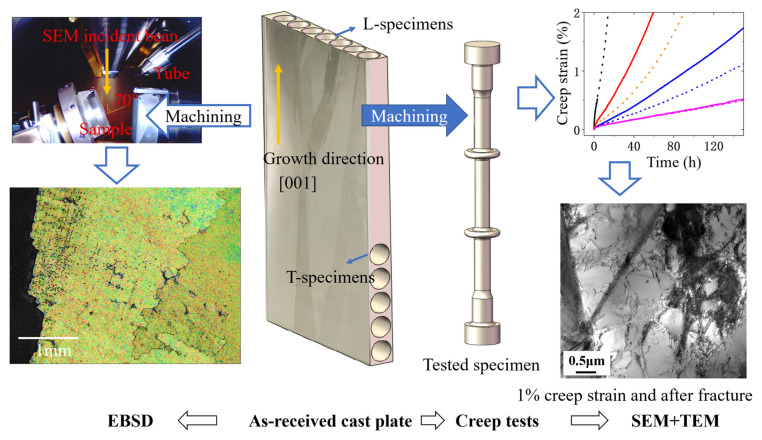
Sampling and testing process diagram.

The casting plates were prepared by directional solidification. The alloy composition is shown in Table 1. The casting plates were subjected to solution treatment and aging using a Titan (H2) vacuum furnace (Ipsen, Souderton, PA, USA). The solution process involved heating at 10 °C/min to 1180 °C and holding for 2 h, then heating at 5 °C/min to 1270 °C and holding for 2 h, followed by argon cooling with a cooling rate of approximately 60 °C/min. The aging process involved holding at 1050 °C for 4 h and then argon cooling with a cooling rate of approximately 40 °C/min. Longitudinal (L) specimens were taken along the [001] growth direction of the casting plate, and transverse (T) specimens were taken perpendicular to the growth direction. Creep tests were conducted at 850 °C under the stress range from 320 MPa to 500 MPa. Three specimens were tested under each condition, with two used to obtain the complete creep curve, and the other removed midway for TEM observation. The sampling and testing process is shown in Figure 1. The creep specimens were designed with a flange to facilitate the installation of the extensometer. The creep tests were performed using an RJ-50 lever-type creep testing machine (Kexin, Changchun, China), with a resistance heating furnace used for heating. The deformation of the specimens over time under the corresponding temperature and stress conditions was collected using an extensometer (Heidenhain, Ulm, Germany).

To better understand the deformation mechanisms of specimens with different orientations during the creep process, the microstructures of the specimens were observed after the creep elongation reached 1% and after fracture. The microstructures were characterized using a Nikon SMZ1270 optical microscope (OM) (Nikon, Tokyo, Japan), a Sigma300 field-emission scanning electron microscope (FESEM) (Carl Zeiss, Cambridge, UK) equipped with an XFlash 6I60 129 eV energy-dispersive spectroscope (EDS) (Bruker, Berlin, Germany) and a Quantax eFlash HR electron backscatter diffractor (EBSD) (Bruker, Berlin, Germany), and a Tecnai F20 transmission electron microscope (TEM) (FEI, Hillsboro, OR, USA). The samples for the OM, FESEM, EBSD, and TEM analyses were taken from the middle of the gauge section of the creep specimens. For the fracture specimens, samples were taken from the area 0–5 mm away from the fracture surface (parallel to the stress axis). The OM samples were prepared by wiping and etching with Kalling’s reagent (100 mL HCl + 100 mL methanol + 5 g CuCl_2_). The fracture surfaces were analyzed using SEM to understand the crack initiation and propagation behavior. The substructures were characterized using FESEM, with an accelerating voltage of 20 kV and an objective aperture of 120 μm. The EBSD samples were prepared using traditional metallographic methods and then lubricated with a 40 nm colloidal silica solution in the polished state to remove strain, without etching. The scanning step size was 0.1 μm. The EBSD data were analyzed using AZtec (Version 2.1) software to generate the grain boundaries (GB) + geometrically necessary dislocation (GND) density. The SEM samples were etched by Kalling’s reagent, the same as the OM samples used to observe γ’ phase. Electro-discharge wire cutting was used to prepare the TEM specimens, which were ground to a thickness of 50 µm using metallographic sandpaper, followed by twin-jet thinning in a solution of 10% HClO_4_ and 90% CH_3_CH_2_OH −25 °C, utilizing Tenupol-5 equipment (Struers, Ballerup, Denmark). Dislocation analysis was performed using the TEM at a voltage of 200 kV.

## 3. Results

### 3.1. Microstructure After Heat Treatment

**Figure 2:**
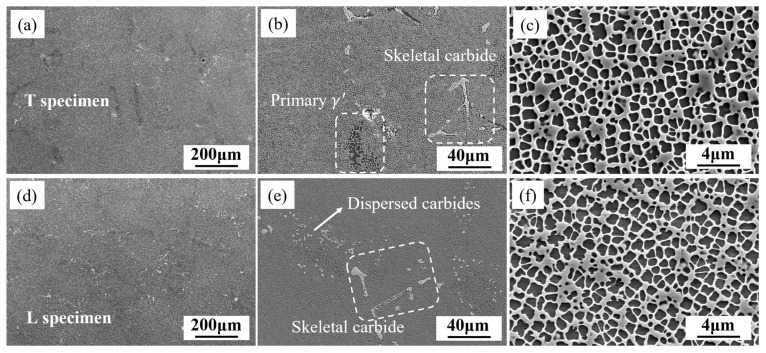
Original microstructure of T and L specimens: dendritic stem (**a**,**d**); interdendritic (**b**,**e**); $\gamma^{'}$ in dendritic stem (**c**,**f**).

The microstructure of the specimens after heat treatment is shown in Figure 2. There is no significant difference in the dendritic structure between the T and L specimens. The γ′ precipitates in the dendritic regions are uniform, with a size of approximately 900 nm and a certain degree of squareness. A small amount of eutectic structure exists in the interdendritic regions, where large-sized γ′ precipitates are present, indicating that the primary γ′ did not completely dissolve during the solution heat treatment. Small-sized blocky carbides and large-sized skeletal carbides are present, with the blocky carbides being dispersed. The skeletal carbides partially dissolved during the solution heat treatment, forming discontinuous short rod-like carbides.

### 3.2. Creep Behavior at 850 °C

**Figure 3:**
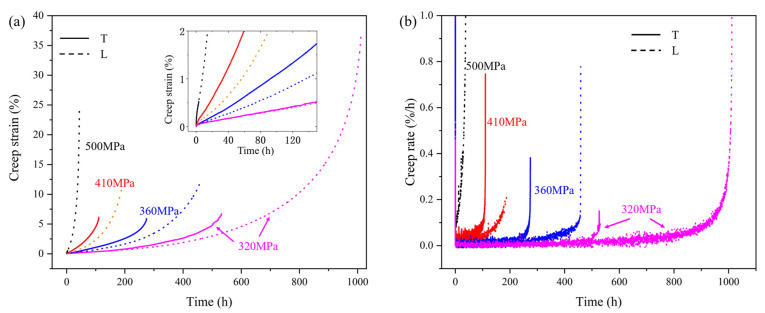
Creep curves (**a**) and creep rate curves (**b**) of T and L specimens under different stresses at 850 °C.

The creep curves of specimens under different stress conditions at 850 °C are shown in Figure 3a, and the creep rates can be obtained by differentiating the strain with respect to time, as shown in Figure 3b. The creep life and steady-state creep rates of the transverse and longitudinal specimens under different stress conditions at 850 °C are shown in Table 2. As the stress increases, the steady-state creep rates of both the transverse and longitudinal specimens accelerate, and their rupture life correspondingly decreases. The longitudinal specimens exhibit better creep properties, characterized by lower steady-state creep rates in the second stage, higher elongation at fracture, and longer rupture life. The creep curve is generally divided into three stages. The first stage is the deceleration creep stage, in which the creep rate rapidly decreases immediately after loading. Therefore, compared with the steady-state creep rate, the rate at the initial stage is very high, as shown in Figure 3b. At 850 °C/320 MPa, the creep rates of the transverse and longitudinal specimens are very close in the first 100 h. After 500 h, the creep rate of the transverse specimen increases, entering the third creep stage earlier. The elongation at fracture of the longitudinal specimens is significantly higher than that of the transverse specimens under all stress conditions, which is close to 40% under the condition of 320 MPa.

### 3.3. Fracture Microstructure After Creep at 850 °C/320 MPa

**Figure 4:**
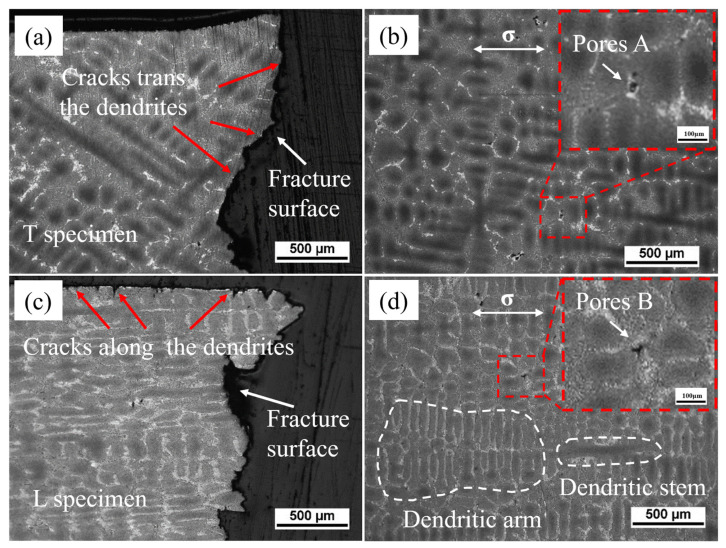
The section morphology near the fracture surface of T specimens (**a**,**b**) and L specimens (**c**,**d**).

In the creep specimens under the condition of 320 MPa, carbides and TCP phases were rarely observed on the fracture surfaces of specimens in both orientations. The fracture surface of the transverse specimen is relatively flat and is shown in Figure 4a. Step-like cleavage planes formed from small facets connected by tear ridges, which indicates that the transverse fracture mode is a mixed mode of cleavage fracture and interdendritic fracture. Near the fracture surface, A-type creep cavities with cracks can be observed, which are rectangular in shape, as shown in Figure 4b. Solidification porosity and homogenization porosity lead to the nucleation of these creep cavities in the interdendritic region. Stress concentration and insufficient plastic deformation in these areas tend to induce cracks [23,24]. The surface of the longitudinal specimen has a large number of secondary interdendritic cracks, and the main crack on the fracture surface propagates along the interdendritic path, as shown in Figure 4c. It can be reasonably inferred that creep micropores preferentially form in the eutectic structure of the interdendritic regions. Near the fracture surface of the longitudinal specimen, some B-type creep cavities with cracks can be observed in the interdendritic regions, which are rhombic and triangular in shape, as shown in Figure 4d. This indicates that after the cracks nucleate near the micropores, they first extend in the direction perpendicular to the stress axis and then propagate along the interdendritic regions when encountering dendrite trunks. When the creep enters the third stage, with the increase in local stress, the cracks can traverse the dendrite trunks horizontally, exhibiting interdendritic fracture characteristics overall.

**Figure 5:**
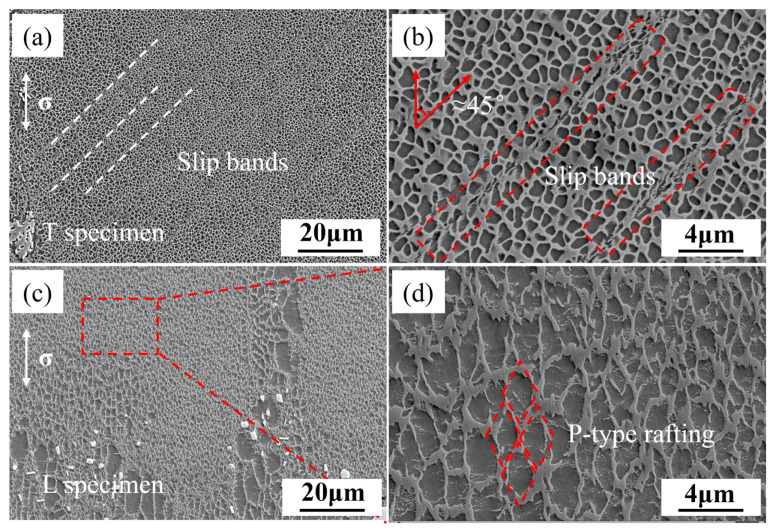
Microstructure of T (**a**,**b**) and L (**c**,**d**) specimens after creep fracture.

Figure 5 shows the microstructure of the alloy after creep fracture at 320 MPa. It can be seen that in the transverse specimen, dislocation slip bands are oriented at 45° to the principal stress direction, and these slip bands directly cut through the γ′. Comparing the rafting conditions of the transverse and longitudinal specimens, it is found that after creep fracture at 850 °C/320 MPa, the γ′ in the transverse specimen still maintains a certain degree of squareness, with no significant coarsening or rafting. In contrast, the γ′ in the longitudinal specimen shows significant coarsening and rafting after creep fracture under the same conditions. Based on the direction of the stress axis and the direction of γ′ coarsening, it can be concluded that it is P-type rafting.

## 4. Discussion

### 4.1. Deformation Mechanisms

**Figure 6:**
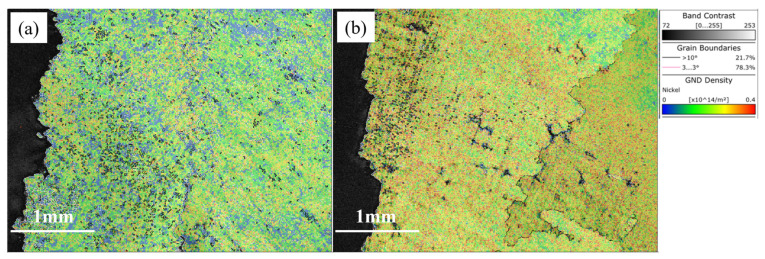
Distribution of GND density near the fracture surface of T (**a**) and L (**b**) specimens.

**Figure 7:**
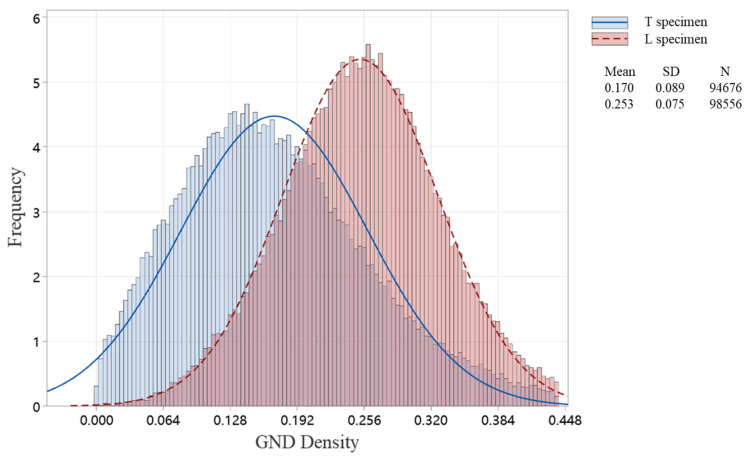
GND density histograms of specimens with different orientations.

Inhomogeneous plastic deformation is a critical factor contributing to fracture and failure in alloy materials. During the deformation process, geometrically necessary dislocations (GNDs) form in the lattice to accommodate local plastic strain gradients. Studying GND density distribution can help analyze the inhomogeneous plastic deformation behavior during creep [25,26,27]. In this study, field-emission scanning electron microscopy equipped with EBSD was used to characterize the GND density of transverse and longitudinal creep specimens; Figure 6 shows the GND density distribution near the fracture surface of both transverse and longitudinal orientations. The GND density distribution data of the two specimens were exported using the AZtec software’s function for exporting raw data of distribution maps. The GND density distribution curves of the two specimens were then plotted using Minitab (Version 17.1.0) software, as shown in Figure 7. It can be clearly observed from Figure 6 and Figure 7 that the GND density of the transverse specimen is relatively lower, and higher near the grain boundaries. The GND density distribution of the longitudinal specimen is rather uniform, with no significant difference in GND distribution near the dendritic boundaries and the dendritic regions. As creep strain increases, the regions with high GND density can expand along newly activated dislocation slip systems or dislocation slip bands, ultimately homogenizing the GND density within the grain. This suggests that longitudinal specimens undergo more uniform creep deformation due to balanced plastic strain distribution.

**Figure 8:**
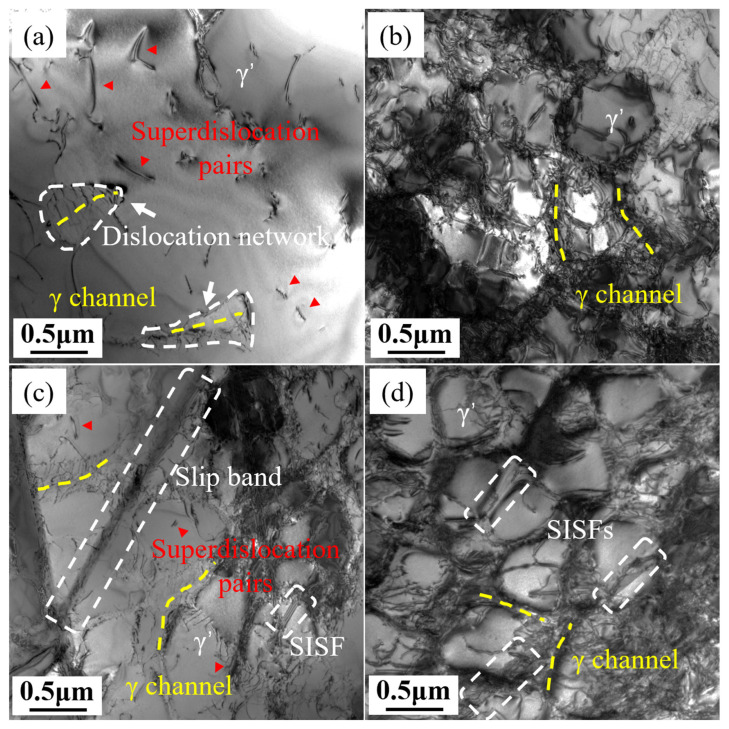
Dislocation structures at 1% deformation: L (**a**); T (**c**); and after fracture: L (**b**); T (**d**).

Figure 8 shows the transmission electron microscope (TEM) observation results of the samples after 1% deformation and fracture. At 1% deformation, dislocation networks can be observed in the γ channel regions of both longitudinal and transverse specimens, and a small number of dislocations can also be seen within the γ′ phase. The dislocation density within the γ′ phase is much lower than that in the γ matrix channels. A large number of dislocations are piled up at the γ/γ′ interface. The dislocation network can relieve the mismatch stress and prevent dislocations from cutting into the γ′ phase, indicating that the γ′ phase acts as a barrier to dislocation motion in the early stages of deformation. Under high stress, dislocations cut into the ordered γ′ phase in the form of pairs of superlattice dislocations. Overcoming antiphase boundary (APB) energy during the cutting process requires higher stress, which increases the deformation resistance of the alloy. However, the cutting process can lead to local atomic disorder, potentially causing strain softening. Figure 8b,d show the different dislocation structures of the longitudinal and transverse samples after fracture. The γ′ phase in the longitudinal sample is more severely cut, with almost no distinguishable intact γ′ phase remaining, and the ordered structure of the γ′ phase has become disordered. In contrast, the γ′ phase in the transverse specimen contains many stacking faults but retains a relatively intact structure. Therefore, under low-temperature/high-stress conditions, the creep deformation mechanisms of both the longitudinal and transverse samples are primarily dominated by the dislocation cutting of γ′. However, the creep properties of the longitudinal and transverse samples show significant differences, especially elongation at fracture. It is necessary to conduct a detailed analysis of dislocation motion, formation of superlattice dislocations, and stacking faults.

**Figure 9:**
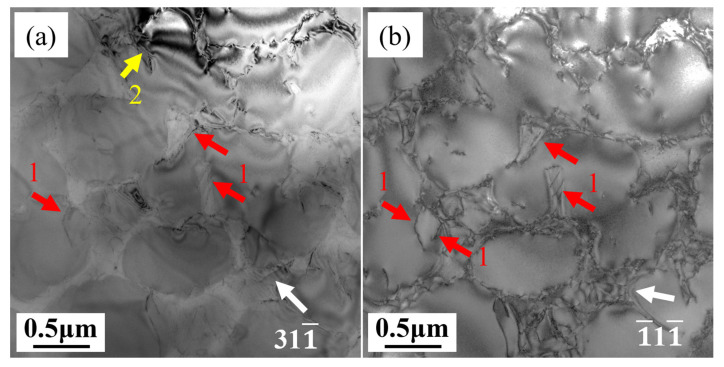

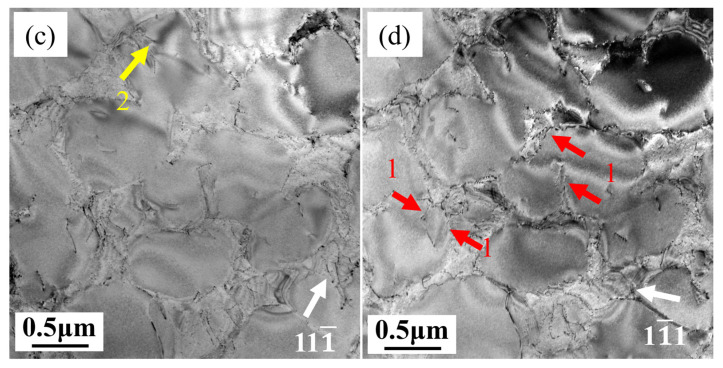
Burgers vector analysis of dislocations 1–2 in the L specimen under different diffraction conditions: (**a**) g: 31$\bar{1}$, (**b**) g: $\bar{1}$1$\bar{1}$, (**c**) g: 11$\bar{1}$, Beam = [011]; (**d**) g: 1$\bar{1}$1, Beam = [$\bar{1}$12].

To investigate the nature of dislocations in longitudinal and transverse creep samples deformed to 1%, a series of two-beam conditions was used under different zone axes and different g-vectors. Figure 9 shows dislocations in the longitudinal sample under the [011] and [$\bar{1}$12] zone axes. Dislocations with different Burgers vectors exhibit visible and invisible characteristics under different g vectors, and Table 3 summarizes the visibility of dislocations 1–2 under different diffraction conditions. According to the extinction rules, the Burgers vectors of these dislocations can be determined as listed in Table 3. In nickel-based superalloys, the γ′ precipitate phase (Ni_3_Al) has an L1_2_ structure, an ordered face-centered cubic structure where Al atoms occupy the corner positions of the cube and Ni atoms occupy face-centered positions. This ordered structure causes dislocations to maintain order during motion, thereby generating antiphase boundaries (APBs) [7,27,28,29,30]. A full dislocation decomposes into two partial dislocations and an APB sandwiched between the two partial dislocations, known as APB shearing [31]. In Figure 9, it can be observed that dislocation 1 in the longitudinal specimen is distributed in pairs, and the Burgers vector of dislocation 1 is determined to be an a/2[101] partial dislocation according to the extinction rules in the two-beam condition. The paired a/2[101] partial dislocations are derived from the decomposition of an a [101] full dislocation. Therefore, it can be inferred that the dislocation reaction occurring in the longitudinal sample due to dislocation shearing of the γ′ precipitates is:

a [101] → a/2[101] + a/2[101] + APB

Other dislocations such as a/2[110] or a/2[10$\bar{1}$] were also found in the longitudinal specimen, indicating that other slip systems are simultaneously activated. In the longitudinal specimen, the stress axis is aligned with the growth direction. In FCC-structured alloys, the close-packed planes are {111}, and the close-packed directions are <110>, so the energy required for dislocations to move in a {111}<110> slip system is the lowest. There are a total of 12 slip systems in {111}<110>. Using the formula of m = cosφcosλ to calculate Schmid factors for these twelve slip systems, eight of them have a Schmid factor of m = 0.408, which is the maximum. Theoretically, these eight slip systems can be activated simultaneously in the longitudinal specimen. According to Figure 8b, in the later stage of creep, the γ′ precipitates in the longitudinal specimen are severely sheared, the L1_2_ ordered structure is disrupted, the alloy softens, and creep enters the third stage. The activation of multiple slip systems is conducive to uniform deformation of the alloy, which is why the elongation at the fracture of the longitudinal specimen is almost 40%.

**Figure 10:**
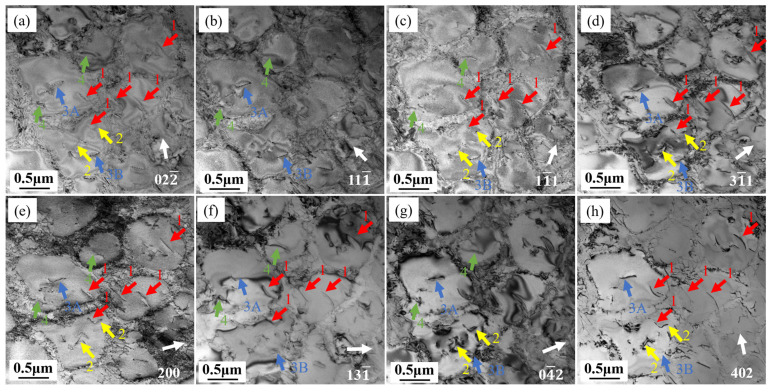
Burgers vector analysis of dislocations 1–4 in the T specimen under different diffraction conditions: (**a**) g: 02$\bar{2}$, (**b**) g: 11$\bar{1}$, (**c**) g: 1$\bar{1}$1, (**d**) g: 3$\bar{1}$1, (**e**) g: 200, Beam = [011]; (**f**) g: 13$\bar{1}$, (**g**) g: 0$\bar{4}$2, (**h**) g: 402, Beam = [$\bar{1}$12].

Figure 10 shows dislocations in the transverse specimen under the [011] and [$\bar{1}$12] zone axes. The dislocations with different Burgers vectors exhibit visible and invisible characteristics under different g vectors, and Table 4 summarizes the visibility of dislocations 1–4 under different diffraction conditions. Interestingly, although dislocations 3A and 3B appear as parallel dislocation lines in transmission electron microscopy, they show different extinction patterns. Under the [011] zone axis, when g vector is $1\bar{1}1$, dislocation 3A is invisible while dislocation 3B is visible; when g vector is 200, dislocation 3A is visible while dislocation 3B is invisible, indicating that dislocations 3A and 3B have Burgers vectors of different orientations. According to the extinction rules, the Burgers vectors of these dislocations are determined as listed in Table 4.

The stress axis direction of the transverse specimen is perpendicular to the growth direction [001], but its secondary orientation on the (001) plane is random. The orientation of the crystal and the stress axis direction together determine the Schmid factors of the various slip systems, which vary under different stress directions. Only when the stress direction of the transverse specimen is [100] or [010], the activated slip systems within a single grain are equivalent to those in the longitudinal specimen, but dislocation motion is still hindered by the columnar grain boundaries. When the stress axis is in other directions, due to its asymmetry, it is difficult to simultaneously maximize the Schmid factors of different slip systems. Therefore, the number of activated slip systems in a {111}<110> slip system of the transverse specimen is far less than that of the longitudinal specimen. When stress is sufficiently high, other slip systems besides the {111}<110> slip system may be activated, such as the {111}<112> slip system [32]. L. Heep et al. [33] calculated the Peach–Koehler slip and climb forces of various slip systems in CMSX-4 single-crystal superalloy at different angles deviating from the [001] direction, indicating that under high-stress conditions, the {111}<112> slip system can be activated. The <112> direction is not an atomically close-packed direction; compared to the <110> direction, it requires overcoming higher lattice resistance. Wu et al. [34] and Bürgers et al. [35] found that <112> dislocations required to cut through the γ′ precipitates can be formed by the reaction of two <110> dislocations within the γ channel. The {111}<112> slip system is more easily activated when the orientation deviates from the [001] direction. Combining the observed dislocation types in the transverse specimen in Table 4 above and the observed SISF in Figure 10, it can be inferred that the dislocation reaction occurring in the transverse specimen when cutting through γ′ precipitates is:

$$a/2[101]\;\to \;a/3[112]+\mathrm{SISF}+a/6[1\bar{2}\bar{1}]$$

$$a/2[101]+a/2[01\bar{1}]\;\to \;a/3[112]+\mathrm{SISF}+a/6[1\bar{1}2]$$

The a/2[101] partial dislocation cuts into the γ′ precipitate and further decomposes into a Shockley partial dislocation. The decomposition process is as follows: an a/2[101] partial dislocation shears from the disordered γ matrix to the L1_2_-ordered γ′ precipitate, forming an a/3[112] Shockley partial dislocation and a superlattice intrinsic stacking fault (SISF) within the γ′ precipitate. The other half of the a/2[101] dislocation with the same Burgers vector, upon entering the γ′ precipitate, generates an a/6[1$\bar{2}\bar{1}$] dislocation while simultaneously annihilating the SISF. The decomposed partial dislocations match the observed dislocation Burgers vectors in the sample.

For the slip bands observed in Figure 5a and Figure 8a, Koehler et al. proposed a cross-slip process as the primary mechanism for the lateral widening of slip lines and the eventual formation of slip bands [36,37,38,39,40]. The slip bands consist of a series of closely packed parallel {111} planes, on which dislocations slide through the γ matrix and γ′ precipitates [41]. Therefore, the slip bands observed in the microstructure of the transverse specimen after fracture are the result of the cross-slip of SISFs on the close-packed planes and their continuous widening.

**Figure 11:**
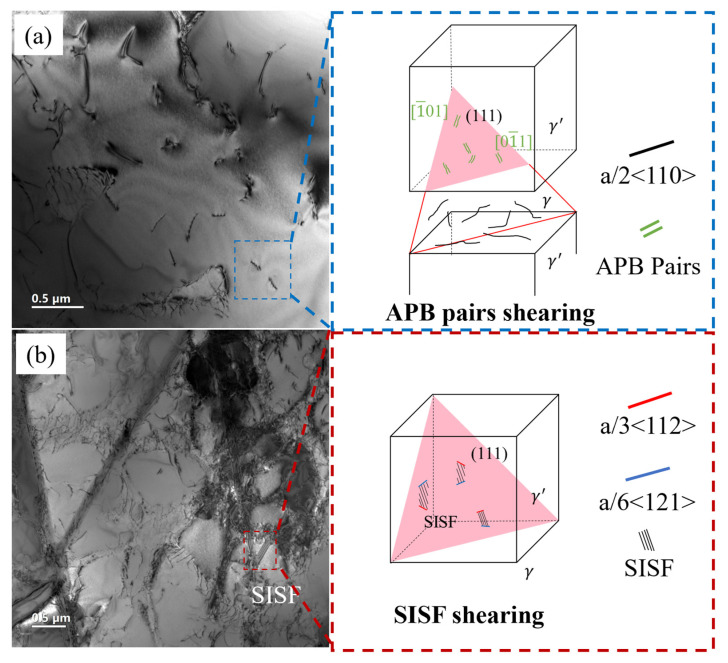
Schematic illustration of the creep deformation mechanisms of L (**a**) and T (**b**) specimens.

Based on the above analysis and discussion, the creep deformation mechanisms of the longitudinal and transverse specimens are summarized and illustrated in Figure 11. Under high-stress conditions at 850 °C, both longitudinal and transverse specimens primarily undergo deformation mechanisms involving dislocation shearing of the γ′ precipitates. The difference lies in the fact that in the longitudinal specimen, APB shearing is predominant, while in the transverse specimen, SISF shearing is the main mechanism. Theoretically, the longitudinal specimen can activate eight {111}<110> slip systems simultaneously. In these slip systems, a full dislocation decomposes into two partial dislocations and an antiphase boundary (APB) that cuts through the γ′. In contrast, the transverse specimen, due to its asymmetry, can only activate a few {111}<110> slip systems. Dislocations pile up in the γ matrix. When the stress is sufficiently high, a {111}<112> slip system is activated, with a/2<101> dislocation cutting into the γ′, forming a/3<112> Shockley partial dislocations and SISFs. According to the dislocation slip characteristics of the transverse specimen of this directionally solidified alloy at 850 °C, when designing hot components, if certain parts of the workpiece are subjected to transverse stress states, more consideration should be given to the risk of fracture failure after relatively short creep deformation.

### 4.2. Creep Microstructural Evolution

**Figure 12:**
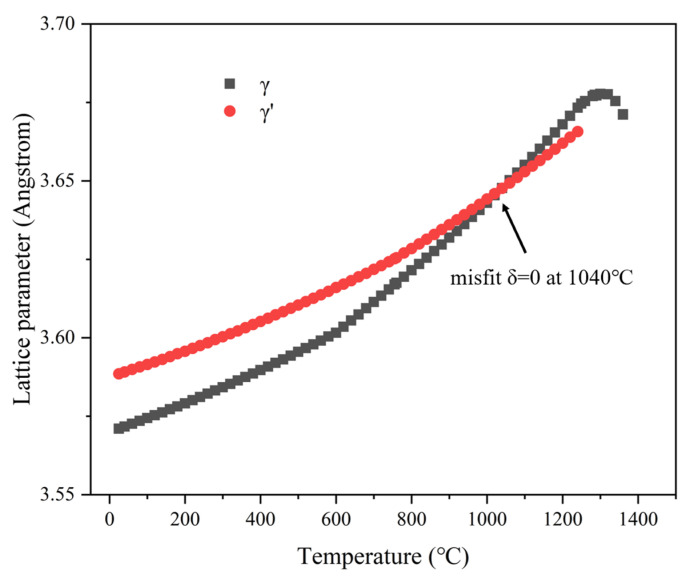
The lattice parameters of γ and γ′ by temperature in the alloy.

The different microstructural evolution patterns of the transverse and longitudinal specimens during the creep process are analyzed. The evolution of the two-phase microstructure during creep can be divided into two steps. The first step is the oriented coarsening of the γ′ precipitates, also known as “rafting”. The second step is the dissolution of the oriented coarsened γ′ phase into the matrix, referred to as “topological inversion” [42,43]. The rafting of the precipitates occurs in the early stage of creep, forming a “wavy” structure. The driving force for rafting comes from the applied stress, the γ/γ′ interface mismatch stress, and the diffusion pipe force of the alloying elements [44]. The variation in the lattice parameters of γ and γ′ with temperature in the alloy is shown in Figure 12. The lattice mismatch between γ and γ′ can be calculated by: [45,46]

(1) $$\delta =\frac{2(a_{\gamma^{'}}-a_{\gamma })}{a_{\gamma^{'}}+a_{\gamma }}$$

where $a_{\gamma }$ is the lattice parameter of the γ phase, and $a_{\gamma^{'}}$ is the lattice parameter of the γ′ phase.

According to Equation (1), the mismatch degree, δ, is positive below 1040 °C and negative above 1040 °C. Therefore, at 850 °C, the mismatch degree is positive. Under tensile stress, the rafting direction of the γ′ phase is parallel to the stress direction, forming P-type rafting.

**Figure 13:**
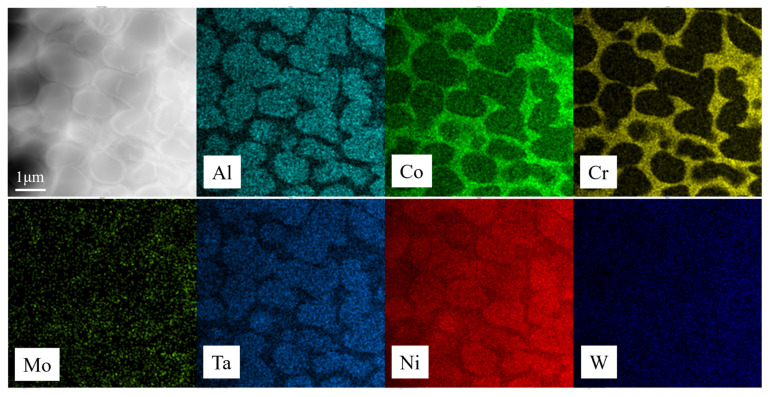
The element distribution in the γ and γ′ phases.

Since the alloy composition is identical, it can be assumed that the γ/γ′ interface mismatch stress is not significantly different between the transverse and longitudinal specimens. Therefore, it is inferred that the different microstructural evolution patterns of the γ′ phase in the transverse and longitudinal specimens are due to differences in the driving force for alloy element diffusion. Transmission electron microscopy equipped with energy-dispersive spectroscopy (EDS) was used to analyze the element distribution of γ and γ′ in the alloy, as shown in Figure 13, and the content of the main alloy elements in the γ and γ′ is given in Table 5.

The diffusion migration rate of each element is analyzed in combination with thermodynamic calculations. Assuming that the volume fraction of the γ phase remains unchanged before and after creep and that the γ′ phase undergoes rafting, the diffusion migration amount of element i, ${∆X}_{i}$ (mol, %), can be expressed as the concentration difference of element i between the γ and γ′ phases, which is:

(2) $${∆X}_{i}=X_{i}^{\gamma^{'}}-X_{i}^{\gamma }$$

where $X_{i}^{\gamma^{'}}$ is the concentration of element i in the γ′ phase, and $X_{i}^{\gamma }$ is the concentration of element i in the γ phase. The values of ${∆X}_{i}$ can be calculated based on the data in Table 5.

Substituting the diffusion migration amount, ${∆X}_{i}$, into Equation (3), the ${∆G}_{i}^{*}$ values for each element can be obtained.

(3) $${∆G}_{i}^{*}=\sum_{j=1,n}\left[{∆X}_{j}{∆G}_{i}^{*j}+\frac{1}{2}\sum_{j,k=1,n(j\neq k)}{∆X}_{j}{∆X}_{k}{∆G}_{i}^{*jk}\right]$$

where ${∆G}_{i}^{*j}$ is the diffusion activation energy of element i in element j, ${∆G}_{i}^{*jk}$ is the diffusion activation energy of element i in element j and k, and ${∆G}_{i}^{*}$ is the diffusion activation energy of element i in the alloy. Substituting ${∆G}_{i}^{*}$ into the following Equation (4) allows the calculation of the diffusion mobility of each element under the given conditions:

(4) $$M_{i}=\frac{1}{RT}exp[\frac{{-∆G}_{i}^{*}}{RT}]$$

where R is the gas constant, and T is the temperature in Kelvin (K). By substituting the calculated ${∆X}_{i}$ values into Equations (3) and (4), the diffusion mobility of each element in the γ′ phase of the alloy at 850 °C can be calculated, as shown in Table 6. It can be seen that the diffusion activation energies and diffusion mobilities of different elements in the γ′ phase of the alloy are different. The order of diffusion mobility from highest to lowest is Al, Ta, W, Mo, Cr, and Co. Among these, Al and Ta are γ′-forming elements, and the diffusion mobility of Al is higher than that of Ta. Therefore, it can be inferred that the Al element is the controlling factor in the morphological evolution of the γ′ phase in the alloy.

Considering the influence of internal stress on the migration of alloying elements, the von Mises stress in the γ channels of the transverse and longitudinal specimens differs significantly under the same external stress conditions. The longitudinal specimen activates more slip systems, and the larger area swept by dislocation slip results in a greater reduction of the von Mises stress within the γ channels. The higher dislocation density leads to more sufficient plastic strain flow, enhancing the directional diffusion of elements and increasing the oriented coarsening rate of the γ′ phase.

Combining the above analysis, it is evident that the Al element is the controlling factor for the rafting of the γ′ precipitates. The longitudinal specimen, with more activated slip systems and sufficient plastic strain flow due to dislocation motion, undergoes P-type rafting. In contrast, the transverse specimen, with difficulty in activating slip systems, shows almost no rafting of the γ′ precipitates.

## 5. Conclusions

Creep tests and microstructural and dislocation structure characterizations were conducted at 850 °C to reveal the creep mechanisms and microstructural evolution patterns of directionally solidified nickel-based alloys in longitudinal and transverse orientations. The key findings include:

1. The longitudinal specimens demonstrated superior creep resistance compared to the transverse specimens under varying stress conditions at 850 °C. This was evidenced by their lower steady-state creep rates and greater elongation at fracture.
2. The creep mechanism in the longitudinal direction involved the activation of multiple {111}<110> slip systems, with dislocations shearing the γ′ precipitates through antiphase boundaries (APBs). In contrast, the creep mechanism in the transverse direction involved the activation of a few {111}<112> slip systems, with dislocations shearing the γ′ precipitates through stacking fault energy (SISF) and forming slip bands at a certain angle to the applied stress axis.
3. Al is the controlling element for the rafting of the γ′ precipitates. The longitudinal specimens, with more activated slip systems and sufficient plastic strain flow due to dislocation motion, underwent P-type rafting. In contrast, the transverse specimens, with limited slip systems, showed almost no rafting of the γ′ precipitates.

However, this study has certain limitations. For example, it does not consider the impact of high-temperature oxidation conditions on the intergranular strength of the specimens. Since 850 °C is in the low-temperature range for turbine blade operating conditions, similar research at higher temperatures is crucial for the application of directionally solidified superalloys. Such work will continue in the future.

## Figures and Tables

**Table 1:** Chemical composition of the casting plates in wt.%.

C	Cr	Co	W	Al	Ta	Mo	Hf	B	Zr	Ni
0.09	7.0	12.0	4.95	5.9	7.0	1.5	1.5	0.015	≤0.1	Bal.

**Table 2:** The creep life and steady-state creep rates of T and L specimens under different stress conditions at 850 °C.

Specimens		320 MPa	360 MPa	410 MPa	500 MPa
T	Creep life, h	533 ± 6	275 ± 4	110 ± 8	4.2 ± 0.8
	steady-state creep rates, %/h	0.0055	0.0224	0.0416	0.1104
L	Creep life, h	1012 ± 59	459 ± 26	191 ± 4	43.6 ± 3
	steady-state creep rates, %/h	0.0042	0.0055	0.0262	0.0988

**Table 3:** The visibility of dislocations under different diffraction conditions of the L specimen.

Beam	g	1	2
[011]	3 $1\bar{1}$	√	√
	$\bar{1}$ $1\bar{1}$	√	×
	$11\bar{1}$	×	√
$[\bar{1}$12]	$1\bar{1}$1	√	×
Burgers vectors b	data	±[101]	$\pm [110]or\;\pm [10\bar{1}]$

Annotations for symbols used in the table: √ for visible; × for invisible.

**Table 4:** The visibility of dislocations under different diffraction conditions of the T specimen.

Beam	g	1	2	3A	3B	4
[011]	$11\bar{1}$	×	×	√	√	√
	$02\bar{2}$	√	√	√	√	√
	$1\bar{1}$1	√	√	×	√	√
	$3\bar{1}1$	√	√	√	√	×
	200	√	√	√	×	√
$[\bar{1}$12]	$13\bar{1}$	√	×	√	√	√
	0$\bar{4}$2	×	√	√	√	√
	402	√	√	√	√	×
Burgers vectors b		±[112]	±[101]	±[121]	$\pm [01\bar{1}]$	$\pm [11\bar{2}]$

Annotations for symbols used in the table: √ for visible; × for invisible.

**Table 5:** The content of the main alloy elements in γ and γ′ (wt.%).

Phase	Cr	Co	W	Al	Ta	Mo
γ	17.39 ± 0.11	19.91 ± 0.13	2.07 ± 0.20	5.74 ± 0.06	0.81 ± 0.20	1.70 ± 0.14
$\gamma^{'}$	3.37 ± 0.06	10.76 ± 0.11	2.02 ± 0.23	14.44 ± 0.09	3.54 ± 0.26	0.57 ± 0.13
$\gamma^{'}/\gamma$	0.19	0.54	0.98	2.50	4.35	0.34

**Table 6:** Diffusion mobility of different alloying elements at 850 °C (m^2^/s).

Elements	Cr	Co	W	Al	Ta	Mo
Mobility	1.80 × 10^−5^	4.37 × 10^−6^	1.05 × 10^−4^	4.00 × 10^−4^	3.13 × 10^−4^	5.48 × 10^−5^

## Data Availability

The original contributions presented in the study are included in the article; further inquiries can be directed to the corresponding authors.
